# Comprehensive evaluation of a pea co-product for piglet nutrition: fibre content, protein digestion, and intestinal barrier function

**DOI:** 10.1093/jas/skaf344

**Published:** 2025-10-06

**Authors:** Maurane Grondin, Messan Kokouvi Djakpa, Frédérique Mayeur-Nickel, Sandra Wiart-Letort, Lucie Le Bot, Frédéric Dessauge, Myriam M -L Grundy

**Affiliations:** INRAE, UMR1348 PEGASE, Institut Agro, 35590 Saint-Gilles, France; INRAE, UMR1348 PEGASE, Institut Agro, 35590 Saint-Gilles, France; INRAE, UMR1348 PEGASE, Institut Agro, 35590 Saint-Gilles, France; INRAE, UMR1348 PEGASE, Institut Agro, 35590 Saint-Gilles, France; INRAE, UR1268 BIA, 44316 Nantes, France; INRAE, UMR1348 PEGASE, Institut Agro, 35590 Saint-Gilles, France; INRAE, UMR1348 PEGASE, Institut Agro, 35590 Saint-Gilles, France

**Keywords:** dietary fibre, gut barrier function, *in vitro* protein digestion, IPEC-J2, paracellular transport, Pea cream

## Abstract

In pig production, weaning is a critical period associated with digestive intestinal disorders, due to the diet and environmental changes. The incorporation of transitional diets with high fibre and protein content represents a promising nutritional strategy to support piglets during the weaning period. This study examined the *in vitro* protein digestion and physicochemical properties of a co-product of interest for piglet at weaning: pea cream. The main objectives were i) to characterise pea cream in detail, focusing on its dietary fibre content, ii) to investigate *in vitro* the hydrolysis of its proteins, and iii) to examine the effect of pea cream digesta on intestinal barrier function using intestinal porcine epithelial cell lines (IPEC-J2). The composition in polysaccharides and the degradation of the pea cell wall were evaluated using biochemical and biophysical methods. The pea proteins from the pea cream were digested using an *in vitro* model of digestion simulating the upper gastrointestinal tract of pigs (based on the INFOGEST protocol). The obtained digesta were detoxified and then applied to IPEC-J2 cells. The results showed that pea cream was rich in dietary fibres, mainly insoluble, and contained approximately 4.6% protein (on an as fed-basis - 76.9% moisture). The *in vitro* protein digestibility of pea cream was high, with a significant release of proteins into the aqueous phase of the digesta earlier on during the digestion process. Microscopy revealed that some proteins remained encapsulated within cell wall fragments even after 6 h of digestion. The exposure of IPEC-J2 cells to detoxified pea cream digesta did not compromise the intestinal barrier integrity, as assessed by the passage of labelled molecules (FD4 and lucifer yellow) and the analysis of tight junction proteins (ZO-1 and occludin). In conclusion, pea cream presents several characteristics that make it a promising candidate for improving piglet weaning. It is a potential source of easily hydrolysable proteins, and its dietary fibres appear to maintain intestinal barrier function in the small intestine.

## Teaser

Pea cream proteins are easily hydrolysed, mostly during the gastric phase. The dietary fibres contained in the digesta maintained intestinal epithelial integrity.

## Introduction

Weaning is a critical period in pig production, during which piglets are submitted to considerable stress, often associated with increased incidence of diarrhoea and reduced growth performance (Tang et al., 2022). These digestive disorders are caused by a number of factors, including abrupt changes of environment, separation from the sow and the transition from a milk diet to a solid diet. Among the strategies available to reduce these disorders, transition feeding plays a key role. The inclusion of dietary fibre in piglet diets can help preserve the integrity of the intestinal barrier (Han et al., 2024). Insoluble fibres, for instance, are known to stimulate peristalsis and improve faecal consistency (Li et al., 2021).

Furthermore, due to environmental and sustainability constrains, there is currently a requirement to use new or unusual source of proteins (notably alternative to animal sources and imported soybeans), for both human and animal nutrition (Parisi et al., 2020; Djuragic et al., 2021; Kumar et al., 2023). Valorisation of co-products of the food industry by developing new products or targeting them for animal nutrition can contribute to the diminution of wastes associated with human food production (Otles et al., 2015; Pinotti et al., 2023). Although, the nutritional composition of certain of these protein sources is known, their behaviour in the digestive tract and the subsequent health impact remain to be determined.

In this context, the notion of protein quality is an essential criterion, where both the composition in amino acids, particularly indispensable, of the proteins and their digestibility ought to be taken into account (Herreman et al., 2020). The digestibility of plant-based protein sources, such as legumes, is usually lower than animal alternatives mainly due to the presence of antinutritional factors, including dietary fibres (Grundy et al., 2016; Sousa et al., 2020; Kaur et al., 2022). Dietary fibres, based on their composition and organisation within the cell walls, can indeed hinder the digestibility of protein and the subsequent process of nutrient absorption and protein metabolism by the organism (Bach Knudsen et al., 2016; Grundy et al., 2022).

*In vitro* models of digestion and absorption are interesting tools that enable scientists to investigate the digestibility of foods while overcoming the limitations associated with studies in humans and animals (Brodkorb et al., 2019; Ji et al., 2022; Hevia et al., 2023). Therefore, with such models a large range of foods, ingredients or food components (e.g., extracted proteins or dietary fibres), can be studied alone or in combination, under well controlled conditions, which is often not possible to do *in vivo* due to safety and/or ethical constraints. This approach permits to identify formulations or diets that can then be tested *in vivo* while providing mechanistic understanding of the process occurring in the gastrointestinal tract (Lentle, 2018).

*In vitro* models commonly found to simulate digestion occurring in swine present some disadvantages compared to the INFOGEST protocol used in the present study (Boisen and Fernández, 1995). One of the major missing steps is the lack of enzyme activity measurement, so it assumes that the activity of the enzyme is consistent regardless of its source or batch, which is not the case (Brodkorb et al., 2019). Moreover, bile salts are omitted and the conditions are not always physiologically relevant. For example, most electrolytes (e.g., calcium, potassium, or magnesium) that make up gastric and pancreatic fluids are absent from these models. However, even though they are present in small quantities, they are essential for optimal enzyme activity and determine interactions between molecules (e.g., the spatial organisation of proteins and polysaccharides).

The integrity of the gut barrier is another parameter that can impact nutrient absorption (Miner-Williams and Moughan, 2016; Chelakkot et al., 2018). It is still unclear how foods and dietary components, such as dietary fibres, influence this barrier function, especially in the upper gut where most of the nutrient hydrolysis and absorption takes place. In this context, cell culture combined with *in vitro* digestion are useful approaches for understanding how these food components are transformed during the digestion process and interact with the intestinal epithelium—mechanisms difficult to study *in vivo* (Kondrashina et al., 2024).

**Figure 1.**
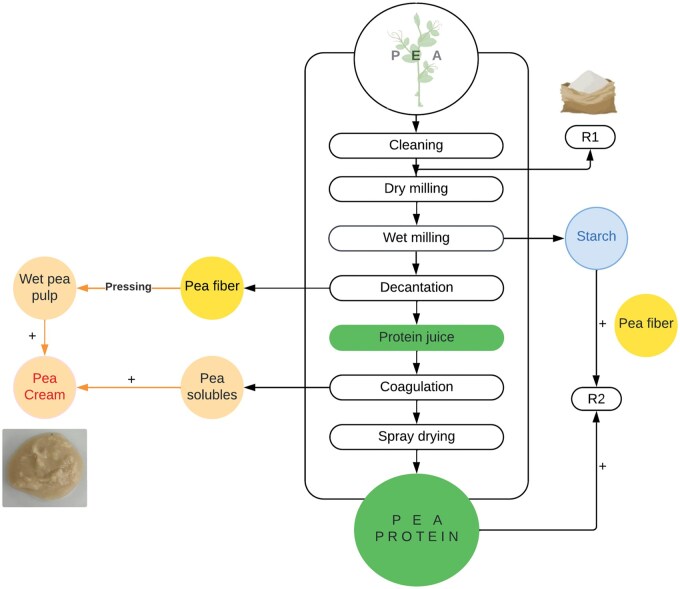
Industrial process used to obtain pea cream and other pea products. Note that R1 (pea flour) is ground from cleaned pea seeds; pea cream contained the wet fraction of pea fibre and other soluble pea compounds (including remaining proteins and starch) generated along the process of extracting protein and starch.

Pea cream is a co-product of the pea starch extraction process that can be a source of proteins and dietary fibres, including oligosaccharides. It is a mixture of pea pulp generated following the milling and decantation of the pea seeds, and the soluble fraction remaining after the protein extraction (see Figure. 1). Our previous work confirmed the barrier role of the cell wall of peas using pea materials in various forms, including pea flour and isolated cells (Grundy et al., 2023; Noel et al., 2024). However, it is unknown if this mechanism takes place in pea cream. Information exists regarding its overall dietary fibre composition but no details are currently available in terms of its polysaccharides content, their physico-chemical properties or their structure (intact and/or ruptured cell wall). According to the literature and our previous studies, pea cell wall is composed predominantly of insoluble dietary fibres, particularly pectin (mainly rhamnogalacturonan) and hemicellulose (mainly xyloglucan) (Brillouet and Carré, 1983; Brummer et al., 2015). Any transformation applied to the pea seed will affect the chemical composition, the physical properties and the health effect of the final product. Dietary fibres could be loss, especially if water is used at some stage of the process (the water-soluble fraction—certain pectin and hemicellulose, or the oligosaccharides—could solubilise during wet milling, see Figure. 1) (Tosh et al., 2010). As a consequence, the behaviour of these dietary fibres in the gastrointestinal tract and the physiological response that follows will differ compare to the original pea ingredient (flour).

The processing of pea as described in Figure. 1 (decantation, pressing, drying) have certainly degraded the cell walls which, in addition to enhancing the protein digestibility of the pea, can produce a series of molecules with potential health effect, such as peptides and oligosaccharides. In addition of being used for animal feeds, pea cream could enter the formulation of foods with targeted functionality. However, the impact of the different types of dietary fibres (polysaccharides solubilised or within cell wall, and the oligosaccharides) on the digestibility of the protein contained in the pea cream, and their impact on the intestinal barrier, has not yet been studied (Perruchot et al., 2025).

Therefore, the aim of this study was to investigate the characteristics and potential of pea cream as a nutritional ingredient to improve piglet digestive health. Specifically, the objectives of this work were: i) to characterise in details the pea cream, with a particular focus on its dietary fibre content, ii) to investigate *in vitro* the hydrolysis of the protein it contains, and iii) to examine the effect of the pea cream digesta on intestinal barrier function using intestinal porcine epithelial cell lines derived from the jejunum of piglets (**IPEC-J2**). Our hypothesis is that pea cream has a dietary fibre and protein (easily accessible to the enzymes) content that is beneficial for piglets. In particular, the structure and organisation of these pea components would enable rapid protein hydrolysis and a positive action of the dietary fibre on the intestinal barrier function.

In order to achieve this, a range of biochemical and biophysical methods were used to evaluate the composition in polysaccharides of the pea cream and the degradation of the pea cell wall following its production. The pea proteins from the pea cream were digested using an *in vitro* model of digestion simulating the upper gastrointestinal tract of pigs (based on the INFOGEST protocol). Then, the obtained digesta were applied to IPEC-J2.

## Materials and Methods

### Materials and chemicals

Pea cream (*Pisum sativum* L.) and other pea materials (protein, fibres, starch and seeds to generate flour and isolated cells) used in this work was provided by Roquette (Vic-sur-Aisne, France). The pea cream was stored at -20°C until further analysis. Pea flour and cells (incubation with NaOH) were obtained as previously described (Grundy et al., 2023; Noel et al., 2024).

Porcine pepsin (P6887, 2326 U/mg of solid), bovine bile extract (B3883), porcine pancreatin (P7545, 3.8 U/mg of solid based on trypsin activity) and fluorescein isothiocyanate–dextran of 4 kDa (**FD4**) were purchased from Sigma (Saint Quentin Fallavier, France). The porcine jejunal intestinal cell line IPEC-J2 was obtained from DSMZ (ACC-701, Braunschweig, Germany; no information was provided by the supplier regarding passage). Lucifer yellow (**LY**), Zonula occludens (ZO-1) monoclonal antibody (mouse) Alexa Fluor^TM^ 594, and Alexa 488 goat anti Rabbit (#A11008) were obtained from Thermo Fisher Scientific (Illkirch-Graffenstaden, France). Occludin monoclonal antibody (rabbit) was purchased from Abcam (#Ab 216327). All other chemicals, solvents and reagents were from Merck (Saint Quentin Fallavier, France) or Thermo Fisher Scientific (Illkirch-Graffenstaden, France).

### Nutritional composition

Pea cream was analysed for protein (Dumas method using a LECO analyser, with a nitrogen conversion factor of 5.4; LECO FP82, Villepinte, France) and starch (AOAC Method 2014.10). Dry matter (oven-dried at 102 °C) and ash were also determined according to standardised methods. Total dietary fibre (**TDF**) content was estimated from the soluble (**SDF**) and insoluble (**IDF**) dietary fibres fractions (method AOAC 991.43).

The composition in neutral sugars of the pea ingredients (flour, fibres and cream) at baseline and the digested pea cream was determined after hydrolysis of polysaccharides into their monomers, followed by individual quantification by chromatography. More specifically, the starch in the pea ingredients (pea cream, pea flour and fibres) were removed via the activity of α-amylase and amyloglucosidase. The alcohol-insoluble residues (**AIR**) were obtained by precipitation with ethanol. The AIR samples were then hydrolysed with sulfuric acid and derivatised into alditol acetates according to (Englyst and Cummings, 1988). In order to determine the contribution of the starch on the glucose content, some pea samples were analysed without hydrolysing the starch. The sugars of the pea ingredients soluble in ethanol were estimated from the ethanol fraction recovered from the production of AIR. Individual neutral sugars were identified and quantified using standards (L-rhamnose, D-fucose, L-arabinose, D-xylose, D-mannose, D-galactose and D-glucose) as well as inositol as internal standard. The alditol acetates were injected onto a GC-FID GC203 (Shimadzu, Marne la Vallée, France) containing a DB225-MS capillary column (30 m × 0.32 mm i.d. coated with, 0.25 µm film thickness; Thermo Scientific). A volume of 1 µL was injected in split mode (ratio 1:50) with an injection temperature of 220 °C. Hydrogen was used as carrier gas at 45 cm/s, the flow rate was 1 mL/min, and the oven temperature was set at 210 °C. Each analysis was performed in triplicate.

### Structural characterisation

The particle size distribution (**PSD**) of the samples was measured at baseline and after digestion (pea cream only) with a laser diffraction particle sizer 3000 coupled to a dispersant unit (Hydro LV) filled with distilled water (Malvern Instruments Ltd, Palaiseau, France). The refractive index of water and pea cream was 1.330 and 1.530 respectively, the absorbance was set at 0.100.

The samples were observed by microscopy with an apotome microscope and the Zen software (Apotome, Zeiss, France) to gain an insight of the microstructure of the pea cream before and after digestion. The technique complemented the PSD data. For the bright field images, the samples were mounted “fresh” on a microscopy slide. The fluorescence imaging was performed after staining the samples with calcofluor white and fast green FCF to identify the dietary fibres (intact cell wall and fragments) and protein, respectively. Images were captured using 10x and 20x objective lenses.

### In vitro digestibility experiments

The pea cream was digested using an adjusted version of the *in vitro* standardised static digestion protocol developed by the INFOGEST consortium (Brodkorb et al., 2019). Briefly, the samples were incubated at 39 °C for 2 min at pH 7 in simulated salivary fluid for the oral phase, 2 h at pH 3 in simulated gastric fluid for the gastric phase, and 2 h or 4 h at pH 7 in simulated intestinal fluids (including bile) for the intestinal phase. The composition of the simulated fluids can be found in the INFOGEST protocol cited above. The quantity of pea cream added corresponded to 50 mg of protein (dry weight basis, which corresponded to 1.08 g of pea cream on the as-fed basis). The samples were incubated without (**B**, bioaccessible proteins) or with (**T**, hydrolysed proteins) enzymes: pepsin for gastric hydrolysis and pancreatin for intestinal hydrolysis. The experiments without enzymes permitted to estimate the amount of protein dispersed into the aqueous phase and potentially available to hydrolysis and absorption (defined here as bioaccessible), while those with enzymes corresponded to the amount of protein hydrolysed. The enzymes activity was stopped, for both the gastric and the intestinal phase, by placing the samples on ice and increasing the pH to 9. They were then centrifuged (4,000 g at 4 °C for 15 min), the supernatant collected and stored at -20°C until further analysis (notably for the IPEC-J2 experiments). The pellet was prepared as described below for protein analysis. Each digestion was performed in triplicate.

### Protein analysis of the digesta

The samples recovered after the gastric or duodenal phases, were centrifuged (4,000 g for 15 min, at 4 °C) and the pellet washed with deionised water on a top of a cell-strainer (Falcon^®^, 40 µm aperture) (Grundy et al., 2022). The washing step permitted to remove the enzymes and other proteins coming from the pepsin, pancreatin, and bile salt preparations. The washed pellets were then dried overnight at 60 °C before being analysed with the LECO combustion analyser as described in 2.2.

The electrophorese profile of the proteins and peptides present in the supernatant was obtained by Sodium Dodecyl Sulphate-Polyacrylamide Gel Electrophoresis (**SDS-PAGE**) using the method described in Grundy et al. (2022).

### Cell culture

The IPEC-J2, passages 3 to 6, were grown in 75 cm^2^ flasks containing Dulbecco’s Modified Eagle Medium/Ham’s F-12 (**DMEM/F12**) supplemented with 10% porcine serum (PS) containing 1% penicillin-streptomycin as previously described (Perruchot et al., 2025). At 80% confluence, cells were seeded on transwell polyester membrane inserts (0.4 μm pore size, 1.1 cm^2^ surface area), placed in 12-well plates at a density of 1x10^5^ cells per cm^2^, and in DMEM/F12 medium supplemented with 5% PS, 1% penicillin-streptomycin, 1% insulin/transferrin/selenium (**ITS**), and 10 µg/mL epithelial growth factor (**EGF**). The cells were left to grow for 14 days in a humidified (95%) incubator at 37 °C under 5% CO_2_. The medium was changed every two to three days and the transepithelial electrical resistance (**TEER**) measured on day 4, 7, 11 and 14 with an Epithelial Voltohmmeter (EVOM3, Friedberg, Germany).

### Evaluation of the impact of pea cream digesta on intestinal permeability

The supernatant obtained following incubation without (samples B) or with (samples T) enzymes were detoxified by 1:10 dilution and heat treatment at 100 °C for 5 min. On day 14 of culture, after exposure for 2 h to either the detoxified digesta or the control (medium only), the passage of FD4 (4 kDa) and LY (521.8 Da) across the epithelial cell monolayers was monitored as previously performed (Perruchot et al., 2025).

For the immunochemistry, the IPEC-J2 were incubated for 2 h with either cell media or detoxified digesta (pea cream samples B and T, 4 h intestinal phase), washed, recovered and fixed with paraformaldehyde (Perruchot et al., 2025). The samples were then incubated for 1 h 30 at 37 °C with either ZO-1 and occludin, diluted 1:100 or 1:400 in PBS-0.2% BSA, respectively. For occludin, Alexa 488 goat anti-rabbit was used as a secondary antibody (1 h incubation at 37 °C, 1:400 dilution). The labelled samples were mounted on microscopy slides with 13 µL of ProLong Gold Antifade Mountant with DNA Stain 4′,6-diamidino-2-phenylindole (DAPI) to stain the nuclei. Images were obtained with a Zeiss Apotome fluorescence microscope using 40X objective. The fluorescence intensity for ZO-1 and occludin was analysed using the ImageJ software. These experiments were repeated three times, on different plates, incubated several weeks apart. Each time two wells were used for each condition, taking 8 images per well.

### Calculation and statistical analyses

Bioaccessible (incubated samples without enzymes) and hydrolysed (incubated samples with enzymes) proteins, expressed in %, were determined using the following equations:


(Eq. 1) $$\mathrm{Bioaccessible}\;\mathrm{or}\;\mathrm{digested}\;\mathrm{protein}\;=\;(\frac{m_{\mathrm{Total}\;\mathrm{original}\;\mathrm{proteins}}-m_{\mathrm{Recovered}\;\mathrm{proteins}}}{m_{\mathrm{Total}\;\mathrm{original}\;\mathrm{proteins}}})*100$$

where m_Recovered proteins_ is the mass in g of protein recovered after digestion and m_Total original proteins_ is the mass in g of protein originally present in the ingredient.

The apparent permeability coefficient of FD4 and LY (*P_app_*, cm.s^−1^) was calculated as follows:


(Eq. 2) $$\mathrm{Papp}=\frac{V}{\left(A\;x\;\mathrm{Ci}\right)}\;x\;\frac{\mathrm{Cf}}{T}$$

where V is the volume in the basal compartment in mL, A is the surface area of the insert, Ci the initial concentration of FD4 or LY in the apical compartment, Cf the concentration of FD4 or LY in the basal compartment, and T the time in second.

The data were analysed using R studio version 4.1.2. For all tests, the significance level was set at P < 0.05 (2 tailed) and all data were expressed as means of triplicates. The differences in protein bioaccessibility and digestibility between the phases, in FD4 and LY diffusion between each condition, and in tight junction proteins (ZO-1 and occludin) intensity were assessed by one-way analysis of variance (ANOVA) followed by Tukey’s post-hoc test.

## Results and Discussion

### Characterisation of pea cream

The composition of the pea cream and other pea ingredients is shown in Table 1. It should be noted that pea cream has a high moisture content, 76.9% (as illustrated in Figure. 3C); which gave a protein content of 4.6% on an as fed-basis as opposed to 19.6% on a dry matter basis (20.8% in pea flour on dry matter basis). Pea cream contained 7.6% of TDF, compared to 17.2% for pea flour. The dietary fibres constitutive of the pea cell walls are primary insoluble accounting for 92.7% of TDF in pea cream. Some starch remained in the pea cream with a residual content of around 17%.

**Table 1.** Chemical composition of the pea cream and other pea ingredients (% on an as-fed basis)

	Pea cream	Pea flour*	Pea fibres*	Pea starch*	Pea protein*
DM (%)	23.1	87.7	95.7	89.3	95.8
Crude protein (%, N × 5.4)	4.6	18.2	6.4	0.54	68.5
Starch (%)	3.9	43.0	34.6	73.5	0.1
TDF (%)^a^	7.6	17.2	49.9	-	-
IDF (%)	7.0	12.1	45.9	-	-
SDF (%)	0.6	5.1	4.0	-	-
Ash (%)	0.5	3.2	3.0	0.1	4.1

* Data obtained from (Grundy et al., 2023).

Pea flour: flour made from pea as described in Grundy et al., 2023; Pea fibres, starch and protein: fibres, starch and protein extracted (by Roquette, the pea ingredients supplier) from pea

**Figure 2.**
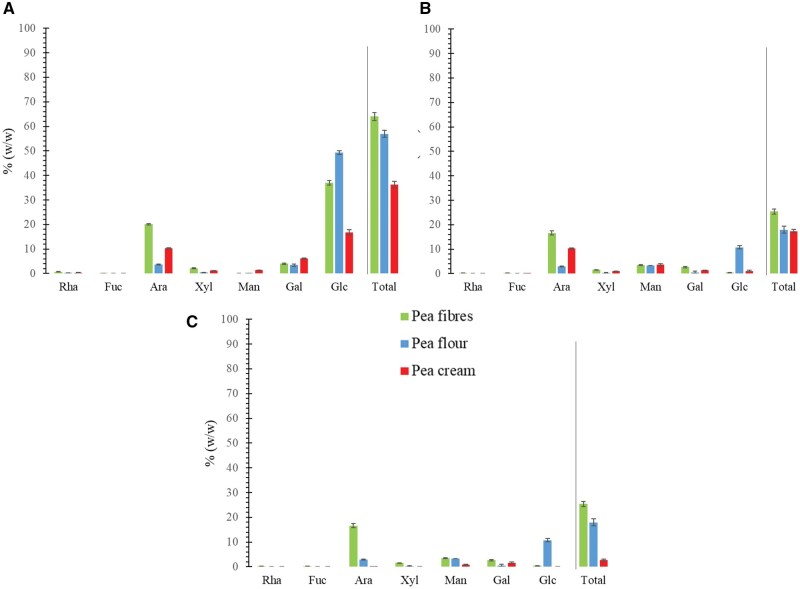
Neutral sugar composition of the pea ingredients at baseline (% dry matter, w/w), without (A) and with (B) starch extraction, and the ethanol soluble fraction (C). Rha, rhamnose; Fuc, fucose; Ara, arabinose; Xyl, xylose, Man, mannose; Gal, galactose; Glc, glucose; Total, Total neutral sugars.

Overall, the pea cream was the sample that contained the least amount of neutral sugars compared to the pea flour and fibres (Figure. 2). The amount of glucose measured is in line with the starch analysis, with pea cream containing less starch granules than the other ingredients (Figure. 2A). This was expected since the pea cream was obtained as part of the extraction process of starch from pea flour. The data from Figure. 2B, where starch was removed by amylase treatment prior to neutral sugar analysis, confirmed these results. According to Figure. 2A, the most predominant sugars composing the pea cream samples were arabinose (10%) and galactose (6%), with cellulose being less abundant (low glucose content, Figure. 2B). Mannose accounted for almost 4% of the pea cream neutral sugars whereas xylose and rhamnose for 1.4 and 0.4%, respectively. These results are overall in agreement with previous studies although pea variety can explain the few discrepancies observed, ie; lower amount of xylose and mannose in our study (Brillouet and Carré, 1983; Brummer et al., 2015). It is likely that the main polysaccharides present in the pea cream cell walls were pectic compounds, as xylose, the main sugar entering in the composition of hemicellulose, was lower (Figure. 2A). A study revealed that pea pectin was indeed rich in arabinan branched to rhamnogalacturonan I and to a lesser extent homogalacturonan was also found (Noguchi et al., 2020). Overall, the polysaccharides found in pea cream were insoluble (Figure. 2C) as showed by the TDF analysis (Table 1). Previous work reported that the oligosaccharides raffinose, stachyose and verbascose (each rich in galactose, glucose and fructose) were found in yellow peas (Han and Baik, 2006; Brummer et al., 2015). However, in the current study, the oligosaccharides produced during the pea cream elaboration process (it is enriched in yeast after the combination of the pea pulp and solubles that could ferment pea cell walls) were not retained during the ethanol precipitation and not detected with the neutral sugar analysis.

**Figure 3.**
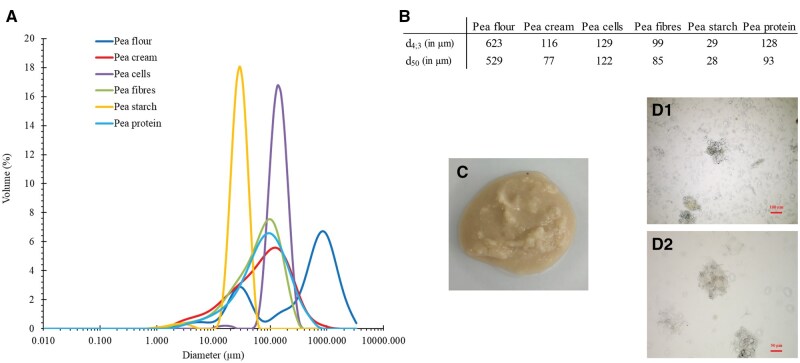
Particle size distribution of the pea ingredients (A), as well as a picture (B) and two microscopy images (C) of the pea cream. Scale bars = 100 and 50 µm, for C1 and C2, respectively.

The PSD of the pea cream showed some heterogeneity with a main peak around 100 µm (median size (d_50_) of 77 µm and a mean size (d_4; 3_) of 116 µm, Figure. 3A and B). From a macroscopic point of view, pea cream looked homogenous with a pasty consistency similar to peanut butter, due to its high moisture content (Figure. 3C). Microscopy images indicated that the particles that make up the pea cream corresponded to starch granules and clusters of cells (Figure. 3D1 and 3D2). Proteins were difficult to identify suggesting that most of them were “loose” (not aggregated as observed in pea protein isolate (Grundy et al., 2023)) or located inside the cells. Given the similarity between the PSD of the fibres, the protein and the pea cream, size measurement was not sufficient to differentiate between these ingredients. In this respect, microscopy complemented this analysis well.

### Digestibility of the pea cream

#### Protein release and hydrolysis

**Figure 4.**
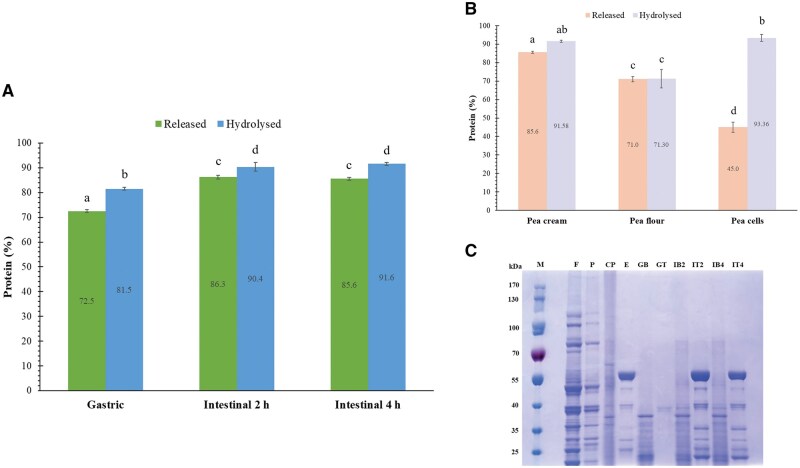
Protein release and hydrolysis during *in vitro* gastrointestinal digestion of pea cream (A) and other pea ingredients (B), and protein profiles by SDS-PAGE (C). M, marker; F, pea flour; P, pea protein; CP; pea cream; E, Enzymes and bile salt (gastric and intestinal phases combined); GB, Gastric phase bioaccessible proteins; GT, Gastric phase hydrolysed proteins; IB2, Intestinal phase bioaccessible proteins 2 h; IT2, Intestinal phase hydrolysed proteins 2 h; IB4, Intestinal phase bioaccessible proteins 4 h; IT4, Intestinal phase hydrolysed proteins 4 h. Protein values in Fig. 4A are expressed as mean ± standard deviation (n = 3). Different letters indicate differences as determined by one-way ANOVA followed by Tukey’s post-hoc test. (P > 0.05). The data presented in Fig. 4B are from Grundy et al., 2022 (pea flour) and Noel et al., 2024 (pea cells).

The hydrolysis of protein was initiated in the gastric phase via the action of pepsin and then in the small intestine via a cocktail of proteases (trypsin, chymotrypsin and carboxypeptidase contained in the pancreatin) (Figure. 4). The results of these reactions lead to the production of peptides of different sizes and amino acids (Figure. 4C). Only small peptides and free amino acids can be absorbed into the bloodstream to be transported at different locations in the body and be metabolised. In order for this to occur, proteins ought to be in contact with the proteases cited above and the products of proteolysis, in absorbable forms (peptides of 3 to 2 amino acids long), released in the “right” site of the gastrointestinal tract, ie the duodenum and the jejunum where most the absorption of proteolysis products happens. However, the structure of the food can prevent this phenomenon, such as dietary fibres within intact cell walls, which encapsulate the protein and thereby prevent the proteases to have access to their substrate (Grundy et al., 2016; Grundy et al., 2023).

Our results demonstrated that most of the protein were bioaccessible (released from the pea cream matrix and present in the aqueous phase of the digesta) and not enclosed within pea cells (72.5%) in the gastric phase, although the intestinal phase led to additional release of the proteins with about 86% being present in the aqueous phase (Figure. 4A). Incubation time did not improve the protein release in the intestinal phase given that 2 h and 4 h gave similar protein values (*P *> 0.05), with a final value of almost 92% of protein hydrolysis after 6 h of incubation (2 h of gastric phase plus 4 h of intestinal phase). The SDS-PAGE analysis confirmed that most of the proteins were hydrolysed during the gastric phase with the exception of a few proteins or peptides of around 40 kD (Figure. 4C). It is difficult to clearly establish if these proteins were still present in the intestinal phase given that some of the proteins present in the pancreatin were of similar size (samples E, IT2 and IT4). The pH of the gastric phase may have degraded and/or aggregated some pea cream proteins which may explain the disappearance of certain protein bands in GB (gastric phase without hydrolysis) compare to baseline (CP), particularly around 50 and 65 kDa. These bands are likely to correspond to vicilin and convicilin (Santos-Hernández et al., 2020; Rivera del Rio et al., 2022). It was noticed that the proteins present in the pea cream were different from those in the pea flour and the extracted pea protein, with the largest proteins being absent from this sample. This was expected given that this co-product originated from protein (and starch) extraction as presented in Figure. 1.

Overall, our results suggested that only a limited amount of protein was encapsulated inside the cells (91.6% of protein hydrolysis). Figure. 4B present the protein results obtained with pea cream, pea flour and isolated pea cells. Pea cell walls appear to be permeable to proteases as showed by the high degree of hydrolysis (over 93%) when the cells are isolated. Whereas for the pea flour the digestion is reduced, due probably to the packing of the cells requiring more time for the enzymes to diffuse through the pea matrix. Indeed, it is well established that macronutrients contained in large particles are slower to hydrolyse (and digest) than in small particles (Grundy et al., 2016; Capuano and Pellegrini, 2019; Lyu et al., 2020; Shurson et al., 2021). A study performed on pea pastes showed differences in protein digestibility based on the cooking methods, with pressure cooking leading to the highest proteolysis compared with ordinary and microwave cooking (Gallego et al., 2020). It is likely that a significant amount of protein remained inside the pea cells, inaccessible to the proteases, even after cooking (only about 20 mg/g of free amino groups for the gastric phase and 74 mg/g for the intestinal phase). Pressure cooking may have resulted in breaking of some of the cell walls, but not all. However, this is only hypothetical as information on the particles present in the samples was lacking.

Therefore, pea cream under our *in vitro* conditions demonstrated high protein release and hydrolysis at the end of the intestinal phase. Unlike pea flour (71% after 6 h of incubation), 4 h of digestion (2 h of gastric phase and 2 h of intestinal phase) was sufficient to reach 91% of protein hydrolysis (Grundy et al., 2023). In order to go further and shed light on the transformation of the pea cream during digestion, a physicochemical characterisation of the digesta was carried out.

#### Evolution of the overall pea cream matrix during digestion

**Figure 5.**
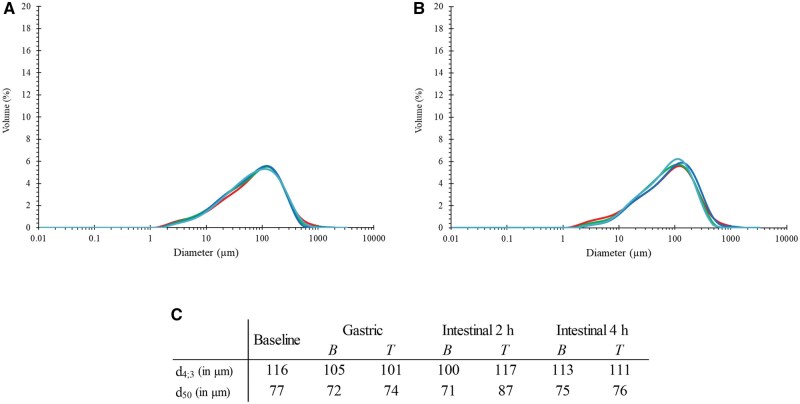
Particle size distribution of pea cream during *in vitro* gastrointestinal digestion. (A) Particle size distribution of pea cream at baseline and after incubation without digestive enzymes (bioaccessible samples). (B) Particle size distribution of pea cream at baseline and after incubation with digestive enzymes (hydrolysed samples) following the gastric phase, 2 h intestinal phase, and 4 h intestinal phase.

**Figure 6.**
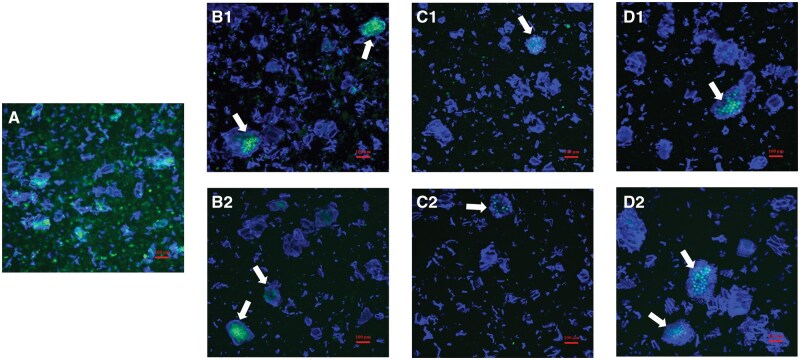
Micrographs of particles present in the pea cream at baseline (A) and recovered in the pellet of digesta after the gastric (B), 2 h intestinal (C), and 4 h intestinal (D) phases, without (1) and with (2) enzymes. Protein are stained green with fast green FCF and cell walls blue with calcofluor white. Note the presence of proteins visible inside some of the pea particles (white arrow). Scale bars = 100 µm.

The washed pellets recovered after incubation, without and with enzymes, for the gastric and intestinal (2 and 4 h) phases, were analysed for the size of the particles they contained. No significant changes were seen in the particles size during incubation without enzymes (Figure. 5A, *P *> 0.05) while some of the small particles disappeared (Figure. 5B, D10 around 16 µm for the intestinal phase compared to 11 µm at baseline, *P *< 0.05). This is likely due to the hydrolysis of the “free” proteins (green coloration outside the pea particles shown in Figure. 6A) as well as to the disappearance of the starch granules which had been hydrolysed by the amylase present in the pancreatin as observed in our previous work (Grundy et al., 2023).

The microscopy images of the particles recovered at different stages of the digestion process of the pea cream (Figure. 6), without or with enzymes, confirmed that most of these particles were dietary fibres (remaining cell wall fragments and cluster of pea cells coloured in blue). The pea protein, stained in green, enclosed inside the large pea particles remained unhydrolyzed even after 6 h of incubation (Figure. 6D2). The digestion performed without enzymes demonstrated that the proteins did not solubilised into the aqueous phase and remained inside the cells. The particles recovered in both conditions appeared similar with proteins still present in Figure. 6D1 and 6D2, the proteases did not reach the protein located in the core of the pea particles (white arrows).

This encapsulation by pea cell wall of nutrients is in agreement with previous work from our lab and other groups (Edwards et al., 2020; Junejo et al., 2021; Grundy et al., 2022; Guevara-Zambrano et al., 2023; Noel et al., 2024). Processing increases the release of the nutrients contained in the plant tissue, however if large particles or intact cell walls remain, as seen with separated cells, nutrient digestibility will be limited (Owusu-Asiedu et al., 2002; Grundy et al., 2016; Oghbaei and Prakash, 2016; Capuano and Pellegrini, 2019; Rivera del Rio et al., 2022).

By definition, dietary fibres are polysaccharides that are not hydrolysed by the host digestive enzymes (Jones, 2014). In some cases, cell walls can be physically degraded during digestion, particularly by solubilisation of its constitutive polysaccharides (Robertson et al., 1997). This scenario does not apply here, where particles similar in size to the ones in the pea cream at baseline (before digestion, d_4; 3_ = 116 µm) were still present at the end of the intestinal phase (d_4; 3_ ∼111 µm). This was to be expected, given that most pea cream dietary fibres are insoluble (Table 1), so they kept their shape and structure throughout digestion, without clear swelling of the cell walls and overall particles. Microscopic images of the particles recovered at the end of the gastric and intestinal phases confirmed this.

Another objective of this study was to monitor the impact of these resistant particles on the intestinal barrier function. To this end, the digesta obtained *in vitro* was used for the experiments carried out on the IPEC-J2.

#### Effect of pea cream digesta on intestinal barrier function

Once hydrolysed, the products of digestion have to cross the intestinal epithelium to reach the host bloodstream and then be metabolised. Studying the diffusion of proteolytic products (peptides and amino acids) through the intestinal epithelium is challenging even using *in vitro* methods. Part of the difficulty lies in the composition of the medium required for cell growth, which must be rich in amino acids. Added to this is the secretion of amino acids and peptides by the cell itself as part of its own metabolism. As a result, indirect methods can be used to evaluate the impact of food components, such as dietary fibres and peptides, on the gut barrier integrity. In this work, the diffusion of labelled molecules of different sizes (4,000 and 522 Da, for FD4 and LY respectively) was assessed and tight junction proteins (ZO-1 and occludin) examined on IPEC-J2 following exposure with pea cream digesta, similar to previous work conducted on pea flour (Perruchot et al., 2025). Before applying the digesta to the IPEC-J2, it is essential to detoxify this digesta to maintain cell viability, among other factors, bile salts and certain peptides can be detrimental to the cells (Kondrashina et al., 2024). For the digested pea ingredients used in this study, it was determined that heat treatment (100 °C for 5 min) and a 1:10 dilution were required to preserve the viability of the IPEC-J2 (Perruchot et al., 2025). Our hypothesis for this part of the work is that if the gut barrier is maintained (healthy not inflamed) the products of proteolysis, of small enough size (free amino acids and peptides of 2 to 3 amino acids), can diffuse (Miner-Williams and Moughan, 2016).

**Figure 7.**
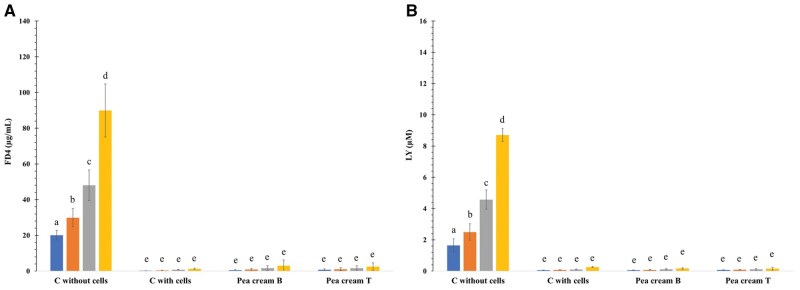
Overtime passage of FD4, in µg/mL (A), and LY, in µM (B) across the IPEC-J2 monolayers after incubation with no cell, medium only (control) or pea cream digesta after 6 h of incubation (without, B, or with, T, enzymes). FD4 and LY concentrations are expressed as mean ± standard deviation (*n* = 3 biological replicates in technical duplicates). Different letters indicate differences as determined by one-way ANOVA followed by Tukey’s post-hoc test (*P* > 0.05).

The first set of experiments demonstrated that neither FD4 or LY diffuse through the IPEC-J2 monolayer (no significant differences between the control and the pea cream digesta, 4 h intestinal, *P *> 0.05), even after 2 h of exposure with the digesta (Figure. 7). The hydrolysis process also had no effect on the diffusion of FD4 (P_*app*__FD4_ = 3.5, 4.7, 3.3 x 10^−8 ^cm.s^−1^ for samples B, T and the control, respectively) or LY (P_*app*__LY_ = 2.2, 2.2, 3.3 x 10^−7 ^cm.s^−1^ for samples B, T and the control, respectively). These results follow the same trends as previous ones, although the current P_*app*_ are overall higher, particularly compared to the well-studied Caco-2 cells and the human small intestine (Rozehnal et al., 2012; Bunchongprasert and Shao, 2020; Perruchot et al., 2025). Few data are currently available in the literature regarding the apparent permeability coefficient of FD4 and LY for the IPEC-J2 cell line, particularly after exposure with digesta (Zakrzewski et al., 2013). On the other hand, LY diffusion was reported to be significantly increased when the IPEC-J2 were challenged with lipopolysaccharides (LPS from *Escherichia coli* serotype O55: B5) (Yan and Ajuwon, 2017; Kiššová et al., 2024).

**Figure 8.**
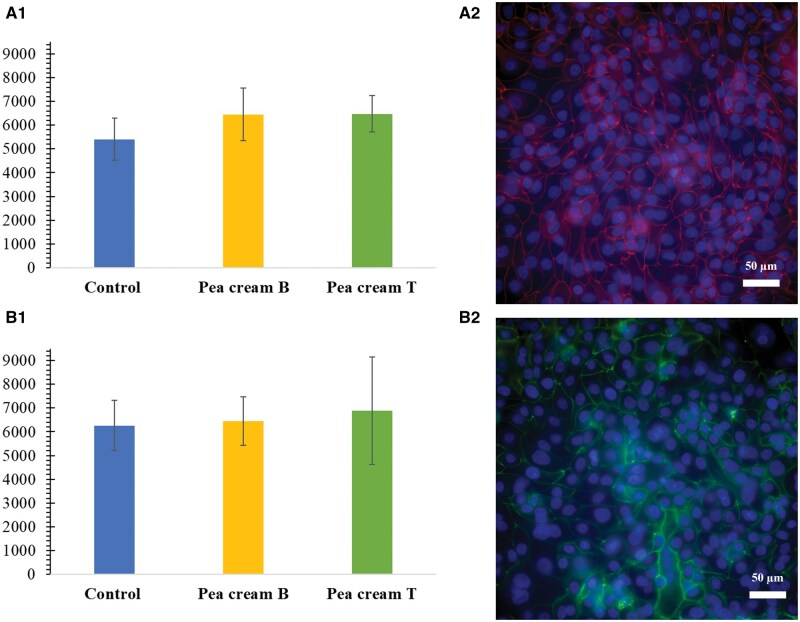
Light intensity measured for ZO-1 (A1) and occludin (B1) as a function of the number of nuclei, and representative images of ZO-1 (A2) and occludin (B2) immunostaining of IPEC-J2 after 2 h of incubation with cell media (control) or pea cream digesta (samples B and T, 6 h of incubation: gastric and intestinal phases). Different letters indicate differences as determined by one-way ANOVA followed by Tukey’s post-hoc test (*P* > 0.05). Scale bars = 50 µm.

As for the tight junction proteins, the outcomes were similar (Figure. 8). Overall, no difference was seen in the intensity per nucleus of ZO-1 and occludin between the control and the pea cream digesta (samples B and T; Figure. 8). Gene expression level of ZO-1 were found to be up-regulated in IPEC-J2 when exposed to fermentation supernatant of inulin and chicory, but not to rye bran, soya hulls or citrus pulp (Uerlings et al., 2020). However, in that study the IPEC-J2 cell line modelled the colonic compartment and the digesta used contained short chain fatty acids (source of prebiotics) that are known to exert a positive impact on the intestinal barrier function (ie, butyrate) (Yan and Ajuwon, 2017). Thus, the mechanisms involved differed from those considered in the present work.

Therefore, these set of experiments (FD4 and LY diffusion and tight junction proteins analysis) demonstrated that the compounds contained in the detoxified digesta, including pea tissue fragments (particles of different sizes, see Figure. S1) and products of digestion, did not compromise the intestinal barrier.

The originally of this research lies on the comprehensive approach employed that included both *in vitro* digestion and a model of jejunal cell to investigate the impact of dietary fibres, from an innovative product, on the digestion process and the gut barrier function. To the best of our knowledge, no other research group investigated the interaction between dietary fibres and intestinal cells in the upper gut (jejunal cells), especially using the IPEC-J2 as a model (GhiselLi et al., 2021; Rodrigues and Failla, 2021). The effect of dietary fibres in the colon has been widely studied however it is still unknow how dietary fibres can influence the absorption of nutrients via their interactions with the mucosa (epithelium and mucins) in the small intestine, main site of digestion and absorption of nutrients (Yang and Zhao, 2021; Xiong et al., 2023; Meldrum and Yakubov, 2025). The findings of this current study shed light on this topic and showed that the integrity of the jejunal cells were not compromised in presence of the pea cream dietary fibres (different in structure and possibly nature from pea flour). Generally, the mechanisms by which dietary fibres can impact intestinal health is by the generation of short chain fatty acids, alteration in intestinal physiology (notably via abrasion), change in mucin secretion, and stimulation of the immune system (Bach Knudsen et al., 2012; Zhang et al., 2023). As an example, pectin has been shown to strengthen the mucosa, including the epithelial cells (Tang and de Vos, 2025). However, the effect observed relied on the structure of the pectin, which illustrates that not all dietary fibres generate the same physiological response, notably because of their specific physico-chemical properties and delivery form (Grundy et al., 2016; Gill et al., 2021; Luo et al., 2022). A recent review performed by Liu et al. (2024) highlighted the various roles of legumes dietary fibres, particularly the insoluble fraction, that stimulates intestinal peristalsis and microbiota diversity. On the other hand, a diet deprived of dietary fibre given to mice led to the degradation of their colonic mucus barrier promoted by the proliferation of mucus-eroding microorganisms (Desai et al., 2016). Similarly, in pig and human a diet low in dietary fibres, particularly insoluble dietary fibres, has a negative impact on the intestinal mucus barrier (Chen et al., 2013; Molist et al., 2014; Stolfi et al., 2023).

Further work should explore the role of the pea cream dietary fibres in stimulating production of mucins by intestinal cells, particularly in the jejunum the main site of nutrient absorption. If the barrier is compromised in the upper gut of piglets, inflammation would occur and the absorption of nutrients will be hindered (Miner-Williams and Moughan, 2016). To address this research question *in vitro* with the IPEC-J2, the presence of mucin and goblet cells should be verified and if possible stimulated. Alternatively, the IPEC-J2 could be co-culture with HT29-MTX as done with Caco-2 cells (Pan et al., 2015). In any case, the characterisation of the IPEC-J2 under a wider range of culture conditions, including co-culture, should be further explored (Nossol et al., 2011; Zakrzewski et al., 2013)

## Conclusions

Pea cream has a potential to be a valuable source of proteins with high digestibility, especially for piglets. The objectives of this study were to i) evaluate the degradation of pea cream, its protein and dietary fibres, during *in vitro* gastrointestinal digestion and ii) monitor the impact of the dietary fibres composing the digesta on the intestinal barrier function. Consequently, this study showed that the dietary fibres in pea cream did not compromise protein hydrolysis (highly bioaccessible as hypothesised) and they seem to have potential health benefits, notably regarding the barrier function in the small intestine. The undegraded particles that will transit to the colon could be a substrate to the microbiota potentially adding other positive health outcome(s). Complementary studies could therefore examine the becoming of these particles and the impact they could have in the distal gastrointestinal tract.

It should be borne in mind that pea cream has a high moisture content, which may present some challenges when formulating diet. Giving the pea cream to piglets at weaning as part of a mash feed could actually facilitate the transition from liquid (milk) and solid feed. Further analysis on the amino acid digestibility could also be performed to adjust the quantity added to the ration and thereby ensure that the piglet requirements on different amino acid are met (protein of high enough quality).

The IPEC-J2 cell line coupled with an *in vitro* gastrointestinal model of digestion provided useful information about the way foods or feeds can be degraded and eventually their nutrients absorbed in the upper gut. As initiated by some researchers, more attention needs to be paid to the culture conditions of the IPEC-J2 in order to optimise the conditions and thereby obtain a physiological environment that permits to study a specific research question (e.g., presence of mucus to study its interaction with food compounds).

## Supplementary Material

skaf344_Supplementary_Data

## Abbreviations

AIR: Alcohol-insoluble residues
B: Bioaccessible proteins
EGF: Epithelial growth factor
FD4: Fluorescein isothiocyanate–dextran of 4 kDa
IDF: Insoluble dietary fibres
ITS: Insulin/transferrin/selenium
LY: Lucifer yellow
PS: Porcine serum
PSD: Particle size distribution
SDF: Soluble dietary fibres
T: Hydrolysed proteins
TDF: Total dietary fibres
TEER: Transepithelial electrical resistance
