# Supplementary material for: Comprehensive evaluation of a pea co-product for piglet nutrition: fibre content, protein digestion, and intestinal barrier function
Source: J Anim Sci. 2025 Oct 6;103:skaf344. doi: 10.1093/jas/skaf344 (PMC12687584; doi:10.1093/jas/skaf344)

**Supplementary material**

Figure S1: Microscopy images obtained with the Apotome following calcofluor white staining for the dietary fibres (cell wall fragments and cell clusters) contained in the detoxified digesta (heat treated and diluted 1:10) for the gastric phase without (GB) and with (GT) enzymes, and the intestinal phase (4 h of incubation) without (IB4) and with (IT4) enzymes. Scale bar = 100 µm.


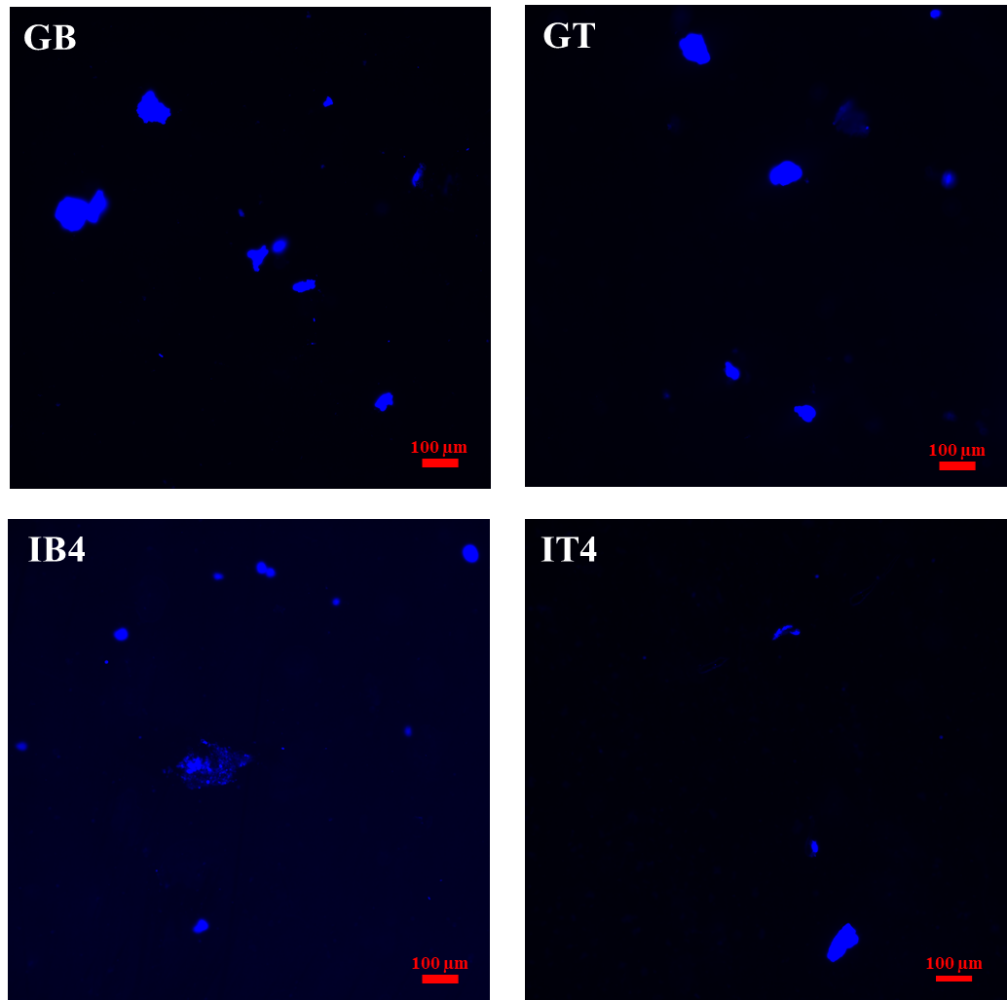

Supplement: skaf344_Supplementary_Data [file skaf344_supplementary_data.docx]
